# Judgement of sleep quality of the previous night changes as the day unfolds: A prospective experience sampling study

**DOI:** 10.1111/jsr.13764

**Published:** 2022-11-27

**Authors:** Nicole K. Y. Tang, Ptolemy D. W. Banks, Adam N. Sanborn

**Affiliations:** ^1^ Department of Psychology University of Warwick Coventry UK

**Keywords:** decision‐making, physical activity, sleep inference, sleep judgement, sleep perception, sleep quality

## Abstract

How we form judgements of sleep quality is poorly understood. Emerging literature suggests that people infer their sleep quality based on multiple sources of accessible information, raising the possibility that sleep quality judgement may evolve as new relevant information becomes available. This study investigated whether people's rating of sleep quality of the night before changes throughout the following day, and what post‐sleep factors are associated with the changes. A prospective experience sampling study of 119 healthy young adults, who completed eight short online surveys interspaced 2 hr apart from 08:00 hours to 22:00 hours. Each survey asked the participants to report total sleep time and sleep quality of the night before, and to provide ratings of current mood, physical and social activity, and pain/discomfort. A memory test was added to the final survey of the day to measure the participants' recall of their first survey responses to sleep quality, as well as total sleep time and mood. The absolute majority (91.1%) of the participants had one or more change in their sleep quality rating across the eight surveys. A similar percentage of change was found for mood rating (100%) but not total sleep time report (20.5%). Memory test in the final survey revealed that the within‐person variations in sleep quality rating were not simply memory errors. Instead, positive physical activity post‐sleep predicted increases in sleep quality rating. Therefore, judgement of sleep quality of the night before changes as the day unfolds, and post‐sleep information can be used by people to infer their sleep quality.

## 1 INTRODUCTION

Sleep is a multidimensional experience (Buysse, 2014). Although polysomnography (PSG) is generally considered the “gold‐standard” for measuring sleep, the perceived experience of sleep—by the sleeper themselves—is just as important, particularly in the process of assessing and treating insomnia disorders (American Academy of Sleep Medicine, 2014; American Psychiatric Association, 2013). Besides self‐reported sleep quantity, there is increasing epidemiological evidence suggesting that self‐reported sleep quality (SQ) is predictive of long‐term physical and mental health outcomes in representative population‐based cohorts (Gadie et al., 2017; Hoevenaar‐Blom et al., 2011; Hublin et al., 2007; Lallukka et al., 2018; Tang et al., 2017). Consistently, in clinical samples that have received insomnia treatment, a 2021 meta‐analysis of 65 randomised‐controlled trials of sleep interventions has found evidence that greater improvements in self‐reported SQ resulted in greater improvements in mental health outcomes (Scott et al., 2021).

Despite its clinical importance, SQ remains an elusive construct without a clear definition (Krystal & Edinger, 2008; Ohayon et al., 2017). Approaches to the assessment of SQ vary across contexts and instruments. When measured with multi‐item questionnaires (e.g. Pittsburgh Sleep Quality Index [PSQI; Buysse et al., 1989], Insomnia Severity Index [Bastien et al., 2001], Sleep Condition Indicator [Espie et al., 2014] and PROMIS Sleep Disturbance Questionnaire [Cella et al., 2010]), SQ is a composite construct reflecting multiple aspects related to SQ over the past month(s), week(s) or day(s). When assessed with daily sleep diaries that ask about sleep of the previous night, SQ is a single qualitative judgement. Typically, the respondent is asked to respond to the question, “How would you rate your sleep quality?”. The term “sleep quality” is usually not defined for the respondent, who is expected to pick a rating on a Likert scale anchored with descriptors such as “very good”, “fair” or “very bad/poor”, based on their sense whether their sleep was good or bad/poor (Carney et al., 2012). It is a “black box” as to *how* people make their SQ judgement and which factor affects their judgement the most.

In the past decades, significant strides have been made in identifying the physiological correlates of sleep that shape people's SQ judgement (ÅKerstedt et al., 1994; Åkerstedt et al., 1997; Della Monica et al., 2018; Keklund & ÅKerstedt, 1997; Svetnik et al., 2020). Amongst experts, the physiological parameters thought to be most relevant to adults' reports of SQ are indicators of sleep continuity (Ohayon et al., 2017). These include the index of sleep efficiency (SE) and the variables involved in the calculation of SE such as sleep‐onset latency (SOL), number and duration of awakening after sleep onset (WASO). There is, however, no consensus on the appropriateness of using sleep architecture variables (e.g. N1, N2, N3, rapid eye movement [REM] sleep %) and nap behaviour (frequency and duration) as indicators of SQ (Ohayon et al., 2017). Recent studies applying machine learning methods have found that besides sleep continuity, PSG‐measured total sleep time (TST) is a significant predictor of self‐reported SQ (Svetnik et al., 2020). However, sleep measures derived from PSG only explained 11%–17% of the variance in quality ratings of prior‐night sleep (Kaplan et al., 2017). SQ judgement may well be determined by some other factors beyond sleep.

Ramlee et al. (2017) examined the relative role of a range of factors thought to define SQ. These factors were not confined to the sleep experience itself, but encompassed time periods before and after sleep. The authors conceptualised SQ judgement as a decision‐making process in which, due to the reduced level of consciousness during sleep, people infer their SQ based on multiple sources of information that are available and accessible. Such a conceptualisation was in line with previously uncovered definitions of SQ from qualitative/mixed methods research (Harvey et al., 2008; Kleinman et al., 2014). In their experiment, Ramlee et al. (2017) simulated the SQ decision‐making process by asking participants to choose between two concrete descriptions of sleep/wake scenarios. Each scenario described a hypothetical self‐reported experience of sleep, stringing together 17 possible determinants of SQ that occur at different times of the day (day before, pre‐sleep, during sleep, upon waking, day after). After repeating this choice exercise over a sufficient number of trials, they quantitatively estimated the relative importance of all included parameters of SQ and examined if these parameters interact with each other. Data from their choice‐based conjoint analysis indicated that SQ judgements were determined by not only what happened during sleep (e.g. TST, WASO, SOL), but also how people felt on waking (e.g. feeling refreshed or not; motivated to get up or not), and what happened during the following day (e.g. mood, social and physical activity engagement; Ramlee et al., 2017). Their findings were consistent with the idea that SQ ratings are inferential in nature, combining memories of the night before and relevant information during the night to make a judgement. They also raised the interesting possibility that people's judgement of SQ may be subject to revision as new information relevant to the decision‐making becomes available. This, if true, will have important implications to assessment and treatment strategies.

The present study therefore investigated whether people's SQ judgement changes as new information relevant to the SQ judgement becomes available. Specifically, we were interested in testing whether people's judgement of SQ (based on sleep from the night just passed) stayed the same when they were asked to provide a rating of the same experience at different times of the next day; and if not, whether any changes in SQ ratings could be explained by variations in mood, engagement in physical and social activities, and pain/discomfort during the day.

We had two key hypotheses. The first hypothesis was that sleep judgement is an inferential process and that people do revise their SQ judgement depending on how their day unfolds. We therefore expected differences between the initial SQ ratings made within 2 hr of waking and those provided subsequently throughout the day, even though the rating was referring to the same sleep episode. The second hypothesis was that any fluctuation in people's ratings of SQ would be associated with physical and social activities in which they have engaged, and their reported levels of pain/discomfort and mood state. Specifically, we anticipated that reports of positive physical and social activities, better mood, and less pain/discomfort during the day would be associated with better ratings of SQ.

## 2 METHODS

### 2.1 Design

**FIGURE 1:**
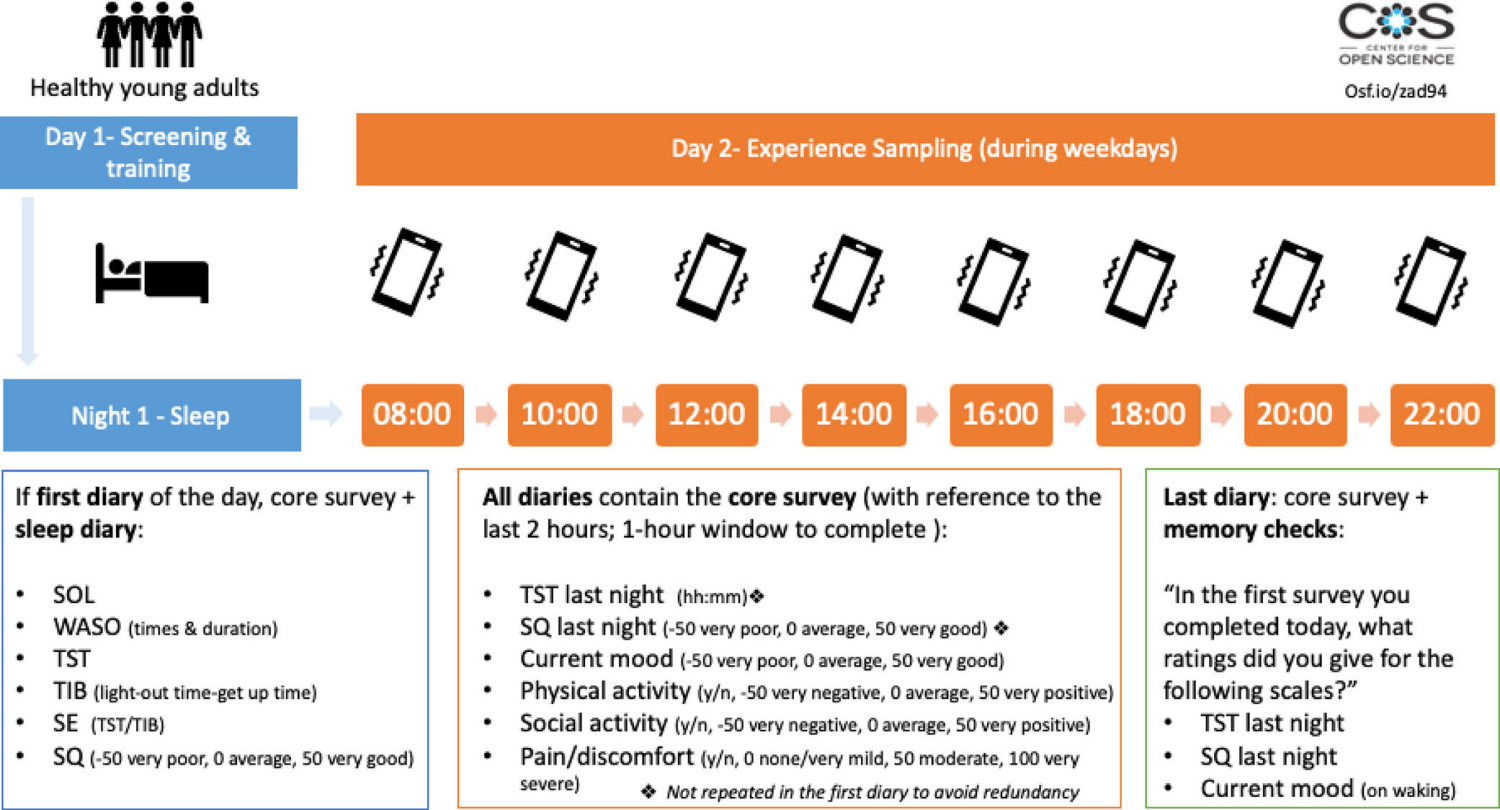
Study design of the 2‐day experience sampling study. SE, sleep efficiency; SOL, sleep onset latency; SQ, sleep quality; TIB, total time in bed; TST, total sleep time; WASO, wake after sleep onset; y/n, yes or no

This was a prospective experience sampling study with a within‐subject design (Figure 1), in which eligible participants were healthy young adults with no diagnosed sleep disorders or known acute sleep disruptions a week prior to taking part in the study. After a pre‐study induction/training session, the participants were asked to sleep as they would typically do in the night, and when they woke up the next day, complete a sleep diary and rate their SQ of the night just passed, their mood, pain/discomfort, and physical and social activities at a maximum of eight different times of the day (08:00 hours, 10:00 hours, 12:00 hours, 14:00 hours, 16:00 hours, 18:00 hours, 20:00 hours, 22:00 hours).

The study protocol had received approvals from the Department of Psychology Research Ethics Committee, University of Warwick, UK. All participants gave informed consent prior to entering the study. The pre‐registration, data and supplementary materials are available on the Open Science Framework (pre‐registration: https://osf.io/zad94; data and analysis code: https://osf.io/z3jhg/).

### 2.2 Participants

One‐hundred and fifty‐eight university students were invited to participate in the study in exchange for course credit. Inclusion/exclusion criteria were: (i) aged 18 years or above; (ii) English‐speaking; (iii) owned a smartphone with a data plan for internet access and an ability to receive text messages (SMS); (iv) no diagnosed medical or psychiatric conditions that impaired their ability to give informed consent and to complete the study; (v) no visual impairments that prevented them from reading/completing the online surveys; (vi) no diagnosed sleep disorders (e.g. sleep apnoea, restless leg syndrome/periodic limb movement disorder, narcolepsy, circadian rhythm sleep disorder); (vii) not experiencing any acute sleep disruption in the week prior to the study (e.g. shift work, jet lag, total/partial sleep deprivation).

One‐hundred and nineteen potential participants attended a pre‐study session to provide informed consent, be screened for eligibility, and be trained for the procedure. Seven were excluded after the session as they did not meet all inclusion/exclusion criteria (*n* = 5; 4 had diagnosed sleep problems, 1 did shift work) or experienced technical issues setting up their mobile phones for the study (*n* = 2). All remaining 112 participants [94% uptake; M_age_ = 19 years (range 18–26 years); female % = 79.5; ethnicity white % = 60.7; M_PSQI_ = 4.41 (range 0–11); chronotype %: evening = 58, morning = 23.2, neither = 18.8] proceeded to complete an experience sampling task the following day.

### 2.3 Procedure

The study comprised a pre‐study session and an experiential sampling task throughout the following day. All participants took part on weekdays to minimise confounds in sleep and other variables associated with weekends (Lenneis et al., 2021).

During the pre‐study session, all participants were given a full overview of the study before providing written consent. The study was described as a “lifestyle study” to minimise demand characteristics. Screening was administered by the researcher using a checklist. Following screening, eligible participants completed a PSQI (Buysse et al., 1989) to indicate their overall SQ in the past month, and a baseline questionnaire that asked single‐item questions about demographics (e.g. age, ethnicity, gender, chronotype: morning, evening, neither). Participants were then offered one‐to‐one training to ensure their understanding of the experience sampling procedure and content of the surveys. They were administered a training survey to ensure their phone received the survey link, through which they were to provide ratings on their SQ, mood, pain/discomfort, and physical and social activities the next day.

The following day, SurveySignal.com was used to send a survey link via SMS to participants at 08:00 hours, 10:00 hours, 12:00 hours, 14:00 hours, 16:00 hours, 18:00 hours, 20:00 hours and 22:00 hours. Participants were explicitly told not to change their sleep and activity routine. If a participant's typical wake time was 09:30 hours and bedtime at 23:00 hours, their first survey would be at 10:00 hours, and only seven surveys were expected of them. For health and safety concerns, the participants were explicitly instructed to not respond to the surveys if they were presented at a time when they were sleeping or when it was simply not safe to do so.

There was a 1‐hr window to complete each survey before the link expired. A reminder text was sent if the participants did not complete the survey after 15 min of the specified time. The survey was administered online using Qualtrics^XM^ survey software.

Each survey took less than 5 min to complete and asked the participants six core questions: (1) “How long did you sleep last night?” (response in hh:mm); (2) “How would you rate the quality of your sleep last night?” (continuous scale: −50 = very poor, 0 = average, 50 = very good); (3) “How would you rate your current mood?” (continuous scale: −50 = very poor, 0 = average, 50 = very good); (4) “Have you engaged in any physical activity in the last two hours?” (yes or no), and if yes, “Was the physical activity a positive or negative experience?” (continuous scale: −50 = very negative, 0 = average, 50 = very positive); (5) “Have you engaged in any social activity in the last two hours?” (yes or no), and if yes, “Was the social activity a positive or negative experience?” (continuous scale: −50 = very negative, 0 = average, 50 = very positive); (6) “Have you experienced any physical pain or discomfort in the last two hours?” (yes or no), and if yes, “How mild/severe was this pain or discomfort?” (continuous scale: 0 = none/very mild, 50 = moderate, 100 = very severe).

All surveys were identical, with two exceptions. First, in the first survey the participants completed on the day, they were asked additional questions about their sleep the night before. These questions established their bedtime, light‐out time, SOL, WASO, wake up time, get up time, and whether they took any medication to help them sleep. Second, the final survey of the day additionally asked the participants to recall their answers to core questions 1–3 in their first survey: (1) “How long did you sleep last night?” (TST); (2) “How would you rate the quality of your sleep last night?” (SQ); and (3) “How would you rate your current mood?” (Mood).

After the participants had completed their last survey at 22:00 hours, they were thanked and debriefed via email the following day.

### 2.4 Data analysis

Missing data were expected for this type of experience sampling study involving frequent surveys at different times of the day. All available data were analysed. The count of observations for each survey were as follows: 08:00 hours (*n* = 50), 10:00 hours (*n* = 88), 12:00 hours (*n* = 105), 14:00 hours (*n* = 108), 16:00 hours (*n* = 104), 18:00 hours (*n* = 100), 20:00 hours (*n* = 92) and 22:00 hours (*n* = 99). In total, we had 746 observations across the 112 participants at the start of the analysis.

Descriptive statistics were used to describe the participants' PSQI scores taken on Day 1 (with reference to the month prior to entering the study), as well as their reporting of TST, SQ and mood throughout Day 2 (i.e. Q1–Q3 of the repeated survey).

The primary interest of the current study was change in participants' SQ ratings throughout the day. Each participant's SQ rating upon waking (i.e. the SQ rating from the first diary of the day) was taken as the baseline, and for each subsequent period the increase (+) or decrease (−) in SQ rating of that period compared with the previous period was used as the dependent measure (SQ change score). As the SQ rating referred to a past event, a non‐zero SQ change score would indicate a change in the participants' evaluation of their sleep the previous night. Outliers (> 3 SDs) in SQ change score were identified and excluded case‐wise in the analysis, leaving 739 observations for the analysis (8 observations: *n* = 24; 7 observations: *n* = 37; 6 observations: *n* = 37; 5 observations: *n* = 11; 4 observations: *n* = 2; 3 observations: *n* = 1).

While not specified in the pre‐registration, to ascertain whether changes in SQ ratings were accessible or inaccessible to participants, and hence a memory error, we first estimated memory error by calculating the absolute change between the first SQ rating and a participant's memory of the first SQ rating, which was taken at the end of the day. We compared this estimate of memory error to the absolute change between the first SQ rating and the SQ rating taken at the end of the day. If the change was not entirely due to memory error, which could reflect an implicit inference process, then the absolute change between the first and last SQ ratings should be larger than the memory error estimate. As a further control, we performed the same analysis for TST and mood. We did not expect participants' experiences during the day to influence their estimate of TST, to the same extent as they would their mood ratings. It was, however, exploratory as to whether the SQ rating versus SQ memory difference would approximate that of TST, mood or neither.

To identify the factors that predict changes in SQ ratings, a linear mixed model was fit with restricted maximum likelihood estimation. The independent variables (or predictors) were mood ratings, pain/discomfort ratings, and responses to the physical and social activity questions, recoding “no” as zero and recoding the rating as either a positive or negative one depending on whether it was a positive or negative experience. In this way, we tested whether mood, pain/discomfort, and physical and social activities that occurred after waking influenced the participants' evaluation of SQ. The full model was: change in SQ rating ~ intercept + physical activity + social activity + mood + pain/discomfort. The fixed effects were reported for each model, and in all of the mixed models we used random intercepts for each participant. We did not use random slopes because the fit was singular even with random intercepts alone. There is no consensus on how to deal with this issue, so we followed one of the recommended solutions: we retained the random intercepts because we theoretically expect random variation across participants (Bolker, 2022). Any observations with missing values for these variables were removed, leaving *n* = 614/739 (83%) to be fit by this model. We set the significance level alpha of the full model to *p* < 0.05, two‐tailed. Calculation of the *p*‐values used Satterthwaite's method. (Satterthwaite, 1946).

The relative importance of each individual predictor was evaluated with both Bayesian Information Criteria (BIC) and Akaike Information Criteria (AIC) scores (Wagenmakers & Farrell, 2004), derived from comparing the following five models: (1) change in SQ rating ~ intercept + physical activity; (2) change in SQ rating ~ intercept + social activity; (3) change in SQ rating ~ intercept + mood; (4) change in SQ rating ~ intercept + pain/discomfort; (5) change in SQ rating ~ intercept. Each of these models was fit by maximising likelihood.

In addition, we included demographic variables in all of the regression analyses as covariates (e.g. age, gender, ethnicity, chronotype, baseline PSQI score) to examine whether these characteristics moderate the effect of physical or social activity, mood, and pain/discomfort on SQ ratings.

## 3 RESULTS

### 3.1 Participants' typical sleep characteristics

The mean global PSQI score reported by the participants as a group was 4.41 (SD = 2.09, median = 4.00), with 34 participants (30.4% of the sample) having a PSQI global score larger than the suggested cut‐off value, 5 (6: *n* = 14; 7: *n* = 14; 8: *n* = 2; 9: *n* = 3; 11: *n* = 1). On average, the participants' typical SOL was 36.94 min (SD = 28.94), TST was 479.38 min (SD = 62.10), TIB was 555.54 min (SD = 83.65) and SE was 87% (SD = 12).

### 3.2 Changes in TST, SQ and mood reporting/rating throughout the day

**FIGURE 2:**
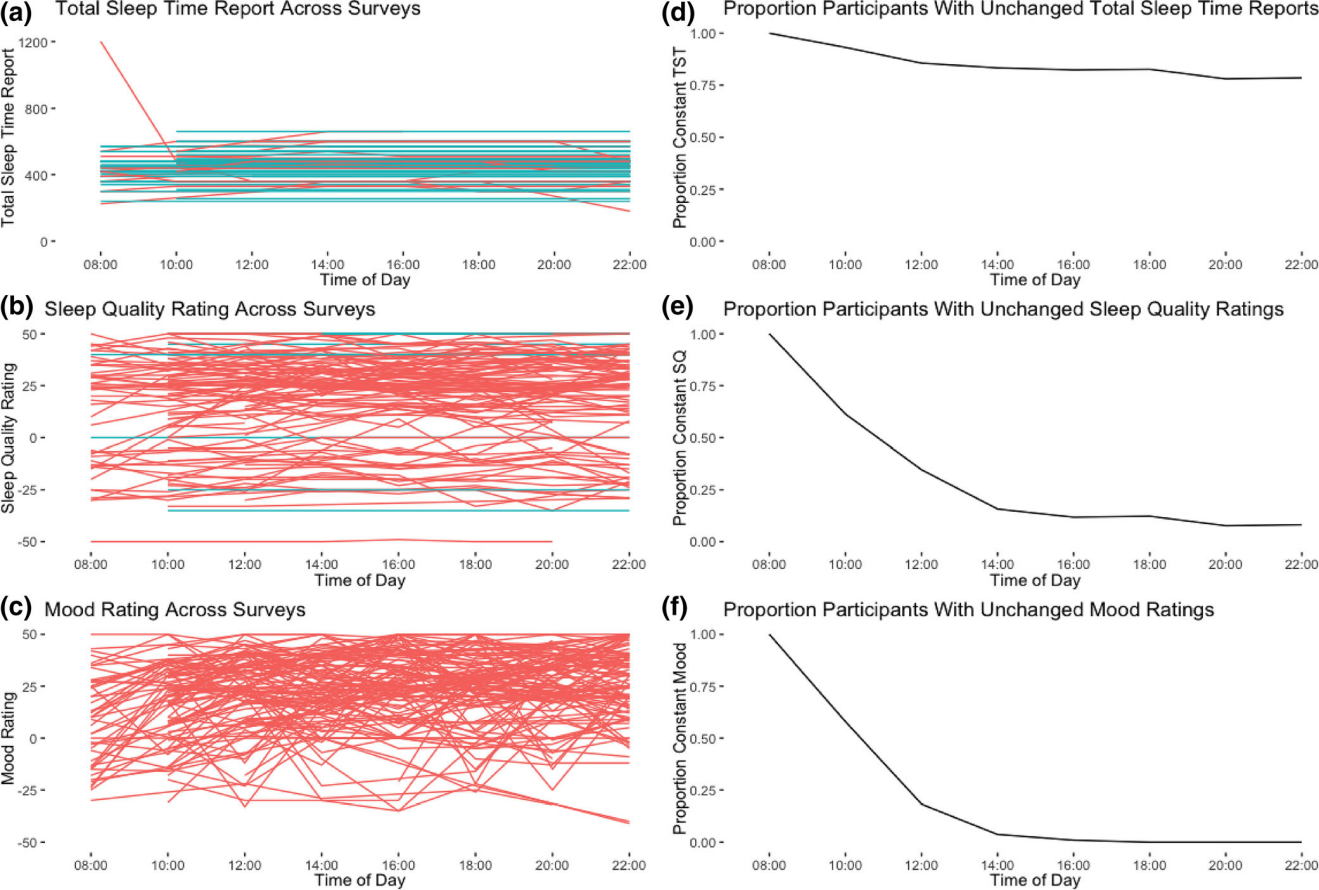
TST reports (a), SQ ratings (b) and mood ratings (c) across the eight surveys of the day, with separate lines for each participant. Blue lines represent participants whose reporting/ratings were constant across all completed reporting periods, whereas red lines represent those whose reporting/ratings changed at least once across reporting periods. (a) The *y*‐axis shows TST reports in minutes. (b) SQ scores are presented (−50 very poor, 0 average, 50 very good). (c) Mood scores are presented (−50 very poor, 0 average, 50 very good). Proportion of participants whose TST reports (d), SQ ratings (e) and mood ratings (f) were constant up through a particular observation time. At 08:00 hours, the lines start at 1 because there is at most a single response per individual, and therefore no variability in a participant's responses. As the day progresses, each participants gives more responses, which decreases the chance that every one of their responses is the same (slight increases in the line are artefacts of calculating the proportions using only participants who responded at that observation point). SQ, sleep quality; TST, total sleep time

Figure 2 depicts the TST reports and ratings of SQ and mood across the eight surveys of the day, with separate lines for each participant. Blue lines represent participants showing no change in their reporting/rating across all reports, whereas red lines represent those with one or more changes.

The TST reports were almost always constant: 89 participants (79.5%) reported exactly the same TST in each completed survey. Despite the SQ ratings being concerned with a fixed past event as the TST reports were, there was a good deal of variability in SQ ratings: only 10 participants (8.9%) reported the same SQ rating for each survey. Ratings of current mood were not constant, as might be expected: 0 participants (0%) reported the same mood rating in each survey.

### 3.3 Are changes in reports/ratings throughout the day due to memory error?

**FIGURE 3:**
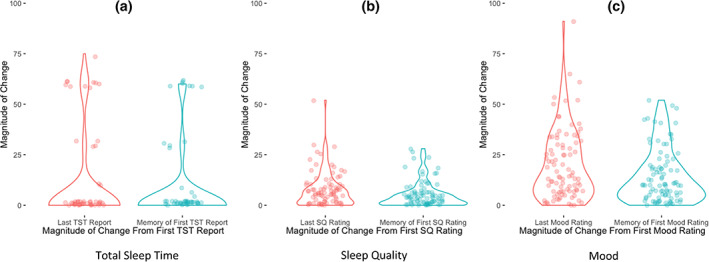
Magnitude of changes between first and last report/rating versus magnitude of changes between actual first report and memory of first report/rating (assessed during the last survey) across TST (a; with 1 extreme outlier excluded), SQ (b) and mood (c). Each data point represents a participant with the shapes (i.e. violin plots) helping to illustrate the density of points: change magnitudes are more prevalent where the shapes are wider. Magnitude of changes between first and last survey = evolution of ratings during the day; magnitude of changes between memory of first survey = memory error. We found that evolution was significantly greater than memory error for SQ and mood. SQ, sleep quality; TST, total sleep time

Figure 3 shows the magnitude of change of TST (a), SQ (b) and Mood (c) between the first survey and the last survey (in red, representing evolution of ratings during the day), as well as the magnitude of discrepancy between participants' first survey reports and their memory of the first survey when asked to recall in the last survey of the day (in blue; representing memory error). When comparing these two magnitudes, there was no significant difference detected for TST (*t*_91_ = 0.72, *p* = 0.47, *d* = 0.08), but changes in SQ and mood throughout the day were significantly higher in magnitude than discrepancies in memory of the initial report/rating throughout the day (for mood: *t*_93_ = 2.81, *p* = 0.006, *d* = 0.29; for SQ: *t*_94_ = 2.32, *p* = 0.022, *d* = 0.24). That is to say, changes in SQ and mood ratings throughout the day were greater than discrepancies in memory throughout the day.

### 3.4 Post‐sleep factors predicting changes in SQ ratings

The mean SQ change score was 0.30 (SD = 5.69, min = −24, max = 25). Based on the full linear mixed model, better physical activity predicted increases in SQ rating, but there was no evidence for mood, social activity, or pain/discomfort having an effect (Table 1).

**TABLE 1:** Post‐sleep factors predicting changes in SQ ratings

Effect of variables in full model	Beta	95% CI	SE	*F*
Mood	0.02	−0.01, 0.05	0.49	2.01
PA	1.17	0.02, 2.31	0.59	3.98*
SA	0.05	−0.96, 1.08	0.52	0.01
Pain	0.01	−0.04, 0.05	0.02	0.14

Abbreviation: PA, physical activity; Pain, pain/discomfort; SA, social activity. Linear mixed model fit by REML. Mixed model ANOVA table (type 3 tests, S‐method). Full model: DeltaSQ ~ Mood + PA + SA + Pain + (1 | ID).

* *p* < 0.05.

Comparing across single‐predictor models with AIC and BIC, physical activity had the best score on both measures, and outperformed the baseline (intercept‐only) model for AIC but not for BIC (Table 2).

**TABLE 2:** Comparing single‐predictor models predicting changes in SQ, using AIC and BIC

Single‐predictor model	df	AIC	BIC
Baseline	3	3977	3990
Mood	4	3976	3994
PA	4	3974	3992
SA	4	3978	3996
Pain	4	3979	3996

Abbreviation: AIC, Akaike Information Criteria; Baseline, intercept‐only model; BIC, Bayesian Information Criteria; PA, physical activity; Pain, pain/discomfort; SA, social activity.

The effect of physical activity also was significant when including any single covariate of age, gender or ethnicity, chronotype, or the hour at which they started the survey (StartHour; Table 3). However, when including PSQI as a covariate, the effect of physical activity was no longer significant. None of the other covariates examined was a significant predictor of SQ change.

**TABLE 3:** Further examination of the role of mood, physical activity, social activity and pain/discomfort in predicting changes in SQ, adjusting for covariates

Model 1		Model 2		Model 3		Model 4		Model 5		Model 6	
Effect	*F*	Effect	*F*	Effect	*F*	Effect	*F*	Effect	*F*	Effect	*F*
Mood	2.00	Mood	2.10	Mood	2.07	Mood	1.88	Mood	1.96	Mood	2.20
PA	3.98*	PA	4.01*	PA	4.01*	PA	3.41	PA	3.98*	PA	4.01*
SA	0.01	SA	0.01	SA	0.11	SA	0.01	SA	0.01	SA	0.04
Pain	0.14	Pain	0.11	Pain	0.22	Pain	0.20	Pain	0.14	Pain	0.14
Age	0.00	Gender	0.21	Ethnicity	0.47	PSQI	0.07	Chronotype	0.17	StartHour	2.59

Abbreviation: PA, physical activity; Pain, pain/discomfort; PSQI, Pittsburgh Sleep Quality Index; SA, social activity. Linear mixed model fit by REML. Mixed model ANOVA table (type 3 tests, S‐method). Full model: DeltaSQ ~ Mood + PA + SA + Pain + Age/Gender/Ethnicity/PSQI/Chronotype/StartHour + (1 | ID). Covariates are presented in the bottom row, with Age in Model 1, Gender in Model 2, Ethnicity in Model 3, PSQI in Model 4, Chronotype in Model 5, StartHour in Model 6.

* *p* < 0.05.

We supplemented the above analyses with three exploratory post hoc *t*‐tests that separately determined whether negative, zero or positive physical activity were associated with SQ rating change. For each test, we selected all the relevant surveys (e.g. those with a negative physical activity rating), computed the mean across surveys for each participant, and participants who did not make any of the relevant ratings were excluded. There were 8 participants who made at least one negative physical activity judgement, 71 who made at least one positive judgement, and 111 who reported no physical activity in at least one survey. Using one‐sample *t*‐tests to compare against no change, there was no statistically significant effect of negative (M = −0.375, *t*_7_ = −0.22, *p* = 0.83, *d* = −0.08) or zero physical activity (M = 0.04, *t*_110_ = 0.16, *p* = 0.87, *d* = 0.02), but there was a significant increase in SQ rating following positive physical activity (M = 1.88, *t*_70_ = 2.69, *p* = 0.009, *d* = 0.32).

## 4 DISCUSSION

Intrigued by the possibility that people's SQ judgement evolves the subsequent day as new relevant information becomes available, we examined changes in SQ ratings when people were repeatedly asked for one, based on their sleep experience the previous night, at different times of the day. The vast majority of the participants in this study (91.1%) revised their ratings, with a non‐zero SQ change score. Whilst the mean magnitude of change was modest, the amount of change varied across individuals, with some demonstrating huge net changes in SQ ratings in both positive and negative directions. Interestingly, the patterns of changes in SQ ratings were not observed for TST reports, but were comparable to changes in people's mood ratings. We also found that the changes in participants' SQ ratings were not simply the result of memory or reporting errors. Instead, physical activities that occurred after the sleep period had an influence on people's SQ ratings during the day, even when the effects of age, gender, ethnicity, chronotype and the start hour of the day were controlled for. Further, the valence of physical activity made a difference in that only positive physical activity had a significant effect on the SQ change score.

These findings provided support to our first hypothesis that SQ judgement is an inferential process, subject to changes that occurred during the day after the sleep period. The evidence also indicated that the changes in SQ ratings are not a confound of circadian rhythm. They were not mere reporting instability or memory errors, because similar changes were not observed for TST reports and the participants were able to recall their first SQ rating of the day with greater accuracy. Our findings provided partial support to our second hypothesis that people's revisions to their SQ ratings are associated with post‐sleep experiences. However, whilst we anticipated that reports of positive physical and social activities, better mood, and less pain/discomfort during the day would be associated with better ratings of SQ, only positive physical activities had a statistically significant effect on changes in SQ ratings. The lack of influence by mood, pain/discomfort, and social activities may be explained by the relative homogeneity of our sample that is generally young and healthy, free of pain and disease. It is possible that, given the developmental stage of our participants (M_age_ = 19 years [range 18–26]), daytime physical functioning was used as the top tell‐tale sign to infer SQ of the night before.

The current study is novel as it sheds new light on the psychological processes influencing our SQ judgement and redefines the meaning of SQ ratings. SQ judgement changing as the day evolves does not mean that SQ is an unreliable measure. It certainly is not a reason to justify the often‐held assumption that ratings of SQ are somehow less scientific or informative than SQ scores calculated from multiple device‐measured sleep parameters, whether they are based on PSG or actigraphy. Instead, it may be more beneficial to consider ratings of SQ and calculated SQ scores as indices of different dimensions of sleep. Our findings revealed that SQ ratings reflect more than just the nighttime sleep experience, but also encompass people's evaluation of their post‐sleep experience. This renewed definition of SQ ratings helps explain why a combination of a large number of PSG‐defined sleep measures explains only up to 17% of variance in SQ ratings (Kaplan et al., 2017).

If SQ encompasses one's evaluation of both sleep and post‐sleep experiences, there are several important implications to sleep assessment, treatment and research. First, in sleep diary studies in which a single rating of SQ is often elicited, variations in SQ ratings over time should be acknowledged and efforts made to standardise the time of sleep diary completion between days within an individual, as well as between individuals within the context of a study. The idea is to ensure that the SQ ratings would be broadly comparable as a judgement made based on similar amounts of information. Not all sleep diaries contain instructions on the timing of completion. Of those that do, like the Consensus Sleep Diary (Carney et al., 2012), the instructions politely suggest that “if possible, the sleep diary should be completed within one hour of getting out of bed in the morning”. Monitoring of adherence is not always possible or practical, but settings could be adjusted in electronic versions of the sleep diary, such that entries will be time‐stamped, and responses not be accepted unless they are provided in the specified time‐frame. This is a common practice in experience sampling studies (Conner et al., 2009). Also, to minimise confounds introduced by variations, it may be necessary to bring the diary completion time closer to the rise time. However, it is an open question when the optimal diary completion time is, without SQ ratings being affected by either the hypnopompic state of consciousness or by the possible influence of post‐sleep activities.

Second, in helping patients to achieve better SQ, there may be synergetic benefits to be reaped by adopting a 24‐hr perspective, actively focusing on both improving sleep experience at night as well as enhancing activity engagement during the day. This is particularly relevant for the management of insomnia disorders and circadian sleep–wake disorders, because reports of clinically significant impairment in functioning are one of the key diagnostic criteria (American Academy of Sleep Medicine, 2014; American Psychiatric Association, 2013). If the engagement in positive physical activity is a factor determining people's judgement of SQ, effects of sleep interventions could potentially be enhanced by incorporating behavioural and exercise interventions (Amiri et al., 2021; Siu et al., 2021), using a hybrid approach. However, future research would need to identify which kinds of physical activity have a therapeutic effect on SQ ratings.

Third, whilst further research is required to identify additional factors influencing SQ judgement, SQ ratings should be viewed as a composite measure. For example, in interpreting the emerging epidemiological link between self‐reported SQ and future physical and mental health outcomes, caution must be applied to avoid viewing this as a pure effect of sleep on physical and mental health. In observing a pattern of declining SQ associated with increasing age, there needs to be an appreciation that other psychosocial factors associated with aging may also be at play. In short, self‐reported SQ can be an indicator of other aspects of healthy functioning.

### 4.1 Strengths and limitations

The current study has strengths and limitations. Its design has a clear temporal order of events, which shows that our participants submitted different SQ ratings at different times of Day 2 despite being asked each time the same question about the same sleep experience on Night 1. Whilst not all participants were familiar with the intensive data collection procedure, we provided each participant with one‐to‐one training and a practice run of the experience sampling procedure to avoid misunderstanding and non‐response. The percentage of missing data in the current study was small (17%) and we had enough observations to power our analyses, as pre‐registered.

In terms of limitations, our sample consisted of healthy young adults, with 30.4% reporting a PSQI global score crossing the clinical sleep disorder cut‐off. Replications with clinical samples of sleep disorders, particularly insomnia disorder, would be required to determine generalisability of the findings reported above, although previous research has found the criteria used to define SQ being broadly similar between normal sleepers and people with insomnia disorder (Harvey et al., 2008; Ramlee et al., 2018). As with other experience sampling studies, our participants were repeatedly presented with the same questions multiple times a day. We cannot preclude the possibility that the participants' response to the questions, as well as their subsequent physical activity, might have been affected by the intensive data collection procedure. Every effort was made to reduce demand characteristics, for example, by presenting the study as a “lifestyle” study, by stating in the instructions that “there are no right or wrong answers”. They were asked to answer the questions based on “how you feel and what seems correct to you right now”, and by not revealing our research focus on SQ ratings until final debriefing. Additionally, when repeating the SQ questions throughout the day, we also repeated the questions about TST and current mood to serve as a distraction as well as a control. We did not see similar changes in TST reports. This can be interpreted as evidence that demand characteristics on the participants to change their SQ rating is likely minimal. Relatedly, it should be noted that participants were not explicitly told to not wear actigraphy or disenable smart phone apps throughout the duration of the study, although they were specifically trained and instructed to respond based on their perception and memory. To reduce demand characteristics, each of the eight daily surveys began with an instruction as follows: “Please note that there are no right or wrong answers. Please answer the following questions based on how you feel and what seems correct to you right now. If for any reason you do not wish to take part in this study, you may withdraw without giving a reason for doing so and without jeopardy.”

We considered the possible influence of sleep inertia on responses when designing the study. To minimise its effect, we instructed our participants to maintain their typical sleep–wake patterns, and anchored the timing of their experience sampling procedure based on their typical sleep–wake patterns. This should help avoid unnecessary undesirable out‐of‐cycle awakening times that accentuate sleep inertia. Given that sleep inertia typically decays within 30 min upon awakening (Trotti, 2017), our participants were only prompted for their first survey 30 min after their typical waking time. If their typical waking time is 09:30 hours, their first survey would be at 10:00 hours, and they have a 1‐hr window to complete the survey before it expires. This should offset the possible effect of sleep inertia on our findings and, consistently, we are assured to see that the starting timing of the survey was not associated with changes in SQ rating (Table 3). Finally, we focused our investigation on factors in the post‐sleep period that may influence people's SQ rating. The fact that we did not investigate factors during sleep did not nullify their direct and indirect influence on SQ ratings. Future research may want to incorporate objective sleep measurements and examine whether the inclusion of post‐sleep factors help to increase the amount of variance in SQ ratings explained by sleep‐related factors.

In conclusion, the complexity of a judgement of SQ may have been under‐recognised. In the absence of accessible memories of sleep, people must rely on a surprisingly large range of information to infer their SQ. This includes internal feelings and external events that occur after the sleep period. This study provided initial evidence that people—given the opportunity—do change their SQ ratings as relevant information emerges. In particular, the engagement of positive physical activity appears to have a significant impact on improving people's SQ ratings. Understanding that SQ ratings reflect more than just the experience of sleep will have implications for our current approaches to SQ assessment, insomnia treatment, and interpretation of epidemiological links between SQ and health outcomes.

## AUTHOR CONTRIBUTIONS

Nicole K. Y. Tang and Adam N. Sanborn developed the study concept, design and pre‐registrations. Testing and data collection were performed by Ptolemy D.W. Banks, with support and supervision from Nicole K. Y. Tang. Adam N. Sanborn and Nicole K. Y. Tang performed the data analysis and interpretation. Nicole K. Y. Tang and Adam N. Sanborn drafted the paper, and all authors provided critical revisions. All authors approved the final version of the paper for submission.

## Data Availability

The data that support the findings of this study are openly available on Open Science Framework. Registration prior to creation of data was done in 2018: preregistration: https://osf.io/zad94; data and materials: https://osf.io/z3jhg/
